# The Expression of Cytokines and Chemokines Potentially Distinguishes Mild and Severe Psoriatic Non-Lesional and Resolved Skin from Healthy Skin and Indicates Different Stages of Inflammation

**DOI:** 10.3390/ijms252011292

**Published:** 2024-10-20

**Authors:** Renáta Bozó, Lili Borbála Flink, Barbara Ambrus, Ameneh Ghaffarinia, Balázs Koncz, Róbert Kui, Rolland Gyulai, Lajos Kemény, Zsuzsanna Bata-Csörgő

**Affiliations:** 1 Department of Dermatology and Allergology, Albert Szent-Györgyi Medical School, University of Szeged, H-6720 Szeged, Hungary; flink.lili.borbala@med.u-szeged.hu (L.B.F.); bata.zsuzsa@med.u-szeged.hu (Z.B.-C.); 2 HCEMM-USZ Skin Research Group, University of Szeged, H-6720 Szeged, Hungary; 3 Synthetic and Systems Biology Unit, Institute of Biochemistry, HUN-REN Biological Research Centre, H-6726 Szeged, Hungary; 4 HCEMM-BRC Systems Immunology Research Group, H-6726 Szeged, Hungary; 5 HUN-REN-SZTE Dermatological Research Group, Hungarian Research Network, H-6720 Szeged, Hungary

**Keywords:** psoriasis, non-lesional skin, psoriasis severity, cytokines, chemokines, resolved skin

## Abstract

In the psoriatic non-lesional (PS-NL) skin, the tissue environment potentially influences the development and recurrence of lesions. Therefore, we aimed to investigate mechanisms involved in regulating tissue organization in PS-NL skin. Cytokine, chemokine, protease, and protease inhibitor levels were compared between PS-NL skin of patients with mild and severe symptoms and healthy skin. By comparing mild and severe PS-NL vs. healthy skin, differentially expressed cytokines and chemokines suggested alterations in hemostasis-related processes, while protease inhibitors showed no psoriasis severity-related changes. Comparing severe and mild PS-NL skin revealed disease severity-related changes in the expression of proteases, cytokines, and chemokines primarily involving methyl-CpG binding protein 2 (MECP2) and extracellular matrix organization-related mechanisms. Cytokine and chemokine expression in clinically resolved versus healthy skin showed slight interleukin activity, differing from patterns in mild and severe PS-NL skin. Immunofluorescence analysis revealed the severity-dependent nuclear expression pattern of MECP2 and decreased expression of 5-methylcytosine and 5-hydroxymethylcytosine in the PS-NL vs. healthy skin, and in resolved vs. healthy skin. Our results suggest distinct cytokine–chemokine signaling between the resolved and PS-NL skin of untreated patients with varying severities. These results highlight an altered inflammatory response, epigenetic regulation, and tissue organization in different types of PS-NL skin with possibly distinct, severity-dependent para-inflammatory states.

## 1. Introduction

Psoriasis is a common immune-mediated, multifactorial, inflammatory skin disease. Chronic plaque-type psoriasis is the most frequently manifesting type, which affects 2–3% of the global population [1,2]. It is characterized by sharply demarcated, red/salmon-pink plaques covered by silver scales. The development of symptoms is influenced by both genetic and environmental factors, irrespective of age or gender. Hyperproliferation of keratinocytes and the infiltration of immune cells have been described as the main characteristic alterations in the psoriatic lesional skin, which contribute to the self-sustaining cycle of inflammation [3]. The clinically lesional and the healthy-looking non-lesional skin areas are well-circumscribed. However, several cellular and extracellular alterations have been described in the non-lesional skin [3,4]. Although psoriasis is primarily a skin disease, it also has systemic effects with several co-morbidities affecting for example the joints and the cardiovascular system. As a result, the patient’s quality of life can significantly deteriorate over the course of the disease. In the last decades, the number of effective treatment options has increased, such as biological therapies that block interleukin (IL)-17A or IL23, but discontinuation of therapy can lead to a recurrence of symptoms in the same areas of the body [3]. Based on recent studies, disease-residual transcriptomic and epigenomic profiles [5], molecular scars [6], and tissue-resident memory cells can remain in the previously lesional skin [7].

Cytokines, including mainly tumor necrosis factor α, interferon-γ, IL17, and IL22, along with antimicrobial proteins like LL-37 [8], growth factors such as epidermal growth factor [9], and chemokines like CCL5 [10] contribute to the development and maintenance of the characteristic psoriatic lesions. Although the exact mechanism of lesion formation is not fully understood, it has been hypothesized that it begins—in a genetically susceptible patient—with the activation of plasmacytoid dendritic cells (pDCs) by various triggers (e.g., LL-37, self DNA fragments, etc.). Subsequently, pDCs activate the myeloid dendritic cells, stimulating Th1, Th17, and Th22 cells. This cascade leads to the hyperproliferation of keratinocytes, aggregation of neutrophils, and the formation of Murno’s microabscesses. Proteases and LL-37 are released by neutrophil granulocytes, the latter of which can be recognized by pDCs to sustain the inflammatory cycle. A considerable number of inflammatory cytokines and chemokines have been shown to partake in these innate and adaptive immune processes, contributing to the formation of the characteristic psoriatic plaques [8].

In psoriasis, tissue remodeling is required for keratinocyte hyperproliferation and immune cell infiltration. Proteases, especially matrix metalloproteinases (MMPs), are actively involved in psoriasis pathogenesis by regulating cell migration, extracellular matrix (ECM) remodeling, angiogenesis, and proinflammatory cytokine activation. In addition to protease inhibitors, cytokines can also influence the functions of MMPs. Increased expression of several MMPs (e.g., MMP1, MMP2, MMP3, etc.) have been described in the psoriatic lesional skin, and besides neutrophils and immune cells, keratinocytes can also produce proteases. Moreover, tissue inhibitors of metalloproteinase 1 and 3 (TIMP1 and TIMP3) exhibit decreased expression in the psoriatic lesional skin, suggesting their role in the regulation of ECM organization during psoriasis pathogenesis [11].

An increasing quantity of evidence indicates that psoriatic non-lesional (PS-NL) skin represents an intermediate state between healthy and clinically lesional skin, with a potential pre-psoriasis phenotype [4,12]. Based on the framework hypothesis, the entire epidermis of psoriatic patients has the capacity to form lesions, and keratinocytes have an inherent capacity for hyperproliferation and abnormal differentiation [13]. Additionally, cellular and extracellular alterations in the psoriatic non-lesional skin can potentially make the skin more susceptible to lesion formation. In the last few years, our research group has also contributed to these observations, which further demonstrate that in addition to the predisposing factors, compensatory alterations can also be found in the psoriatic non-lesional skin, that contribute to the maintenance of the asymptomatic, non-lesional state. The balance of these predisposing and protective factors can prevent keratinocyte hyperproliferation and immune cell infiltration in the non-lesional skin [14]. Abnormalities of the ECM have also been observed in non-lesional skin, especially at the dermal–epidermal junction region, which manifests in the presence of micro wounds [15], and keratinocytes’ special wound-healing-like phenotype [16].

Previously lesional psoriatic-resolved skin is a particular type of healthy-looking non-lesional skin of patients undergoing local or systemic therapy. Recent studies suggest that molecular scars and local memory remain at these skin sites, and provide the basis for recurrent lesion formation [6]. Certain alterations in non-lesional skin, such as the cell cycle inhibitor p27/Kip1 and the Forkhead-box protein O 1 have shown expression patterns dependent on PASI (Psoriasis Area and Severity Index) compared to healthy skin [17]. Disease severity can also influence the expression of MMPs and protease inhibitors in the serum of psoriatic patients [11], indicating the consideration of disease severity during the examination of psoriatic samples. Proteomic studies mainly compare clinically lesional psoriatic skin with healthy or non-lesional skin [18]. However, a limited number of proteomic analyses are available that compare untreated psoriatic patients’ non-lesional skin with the skin of healthy individuals, and disease severity is often not taken into account in these analyses.

In this study, we aimed to explore potential new regulatory mechanisms of tissue organization that can influence the maintenance of the uninvolved state. Therefore, we compared the levels of different cytokines, chemokines, proteases, and protease inhibitors between healthy and non-lesional psoriatic skin from patients showing different disease severities. The levels of cytokines and chemokines were compared between healthy and resolved skin as well. Our results suggest distinct cytokine and chemokine signaling between the non-lesional psoriatic skin of untreated patients with different severities and resolved skin. These results indicate an altered inflammatory response and epigenetic regulation in different types of non-lesional skin with potentially different, severity-related para-inflammatory conditions.

## 2. Results

### 2.1. Variantly Severe Psoriasis Patients Have Different Expression Patterns of Cytokines, Chemokines, Proteases, and Protease Inhibitors in Their Non-Lesional Skin

**Figure 1:**
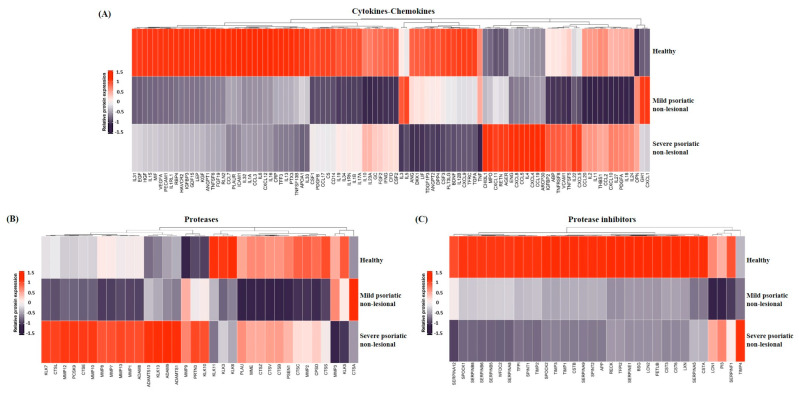
Differential expressions of cytokines/chemokines, proteases, and protease inhibitors between healthy and non-lesional skin of psoriatic patients with mild and severe disease. The levels of cytokines/chemokines (**A**), proteases (**B**), and protease inhibitors (**C**) were compared in whole skin punch biopsies from healthy donors and from non-lesional skin of untreated psoriatic patients. For mild psoriasis, the mean Psoriasis Area and Severity Index (PASI) was 9.06, while for severe psoriasis, the mean PASI was 28.93. Each group utilized *n* = 3 pooled protein extracts. Pixel densities of the different proteins were determined by the Image Studio software 6.0 (LI-COR Biosciences, Lincoln, NE, USA) and presented as relative protein expression. In the visualization, the steel blue color corresponds to the lowest expression, red indicates the highest expression, and white represents medium expression levels of the given protein. Proteins are indicated by gene identifiers and listed in Appendix A Table A1.

To explore whether cytokines, chemokines, proteases, and protease inhibitors belong to a common regulatory pathway, their expressions were analyzed in mild and severe PS-NL versus healthy skin. Based on relative expression data, the level of cytokines, chemokines, and proteases mainly decreased in mild PS-NL versus healthy skin. In contrast, in the non-lesional skin of patients with severe symptoms, several cytokines, chemokines, and proteases showed higher expression than healthy and mild PS-NL skin (Figure 1A,B). Protease inhibitors showed a decreasing expression pattern in both mild and severe PS-NL skin compared to healthy skin (Figure 1C).

### 2.2. Differentially Expressed Proteins (DEPs) Were Related to Hemostasis and Immune Regulation in Non-Lesional Versus Healthy Skin

**Figure 2:**
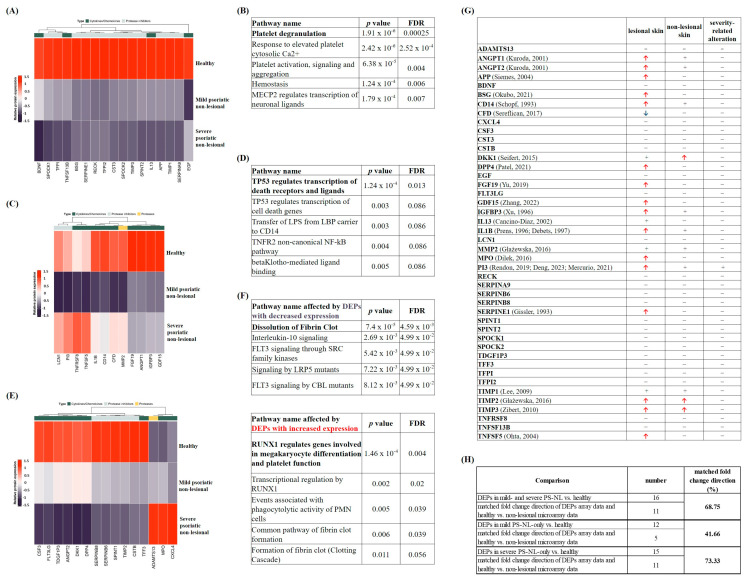
Differentially expressed proteins (DEPs) and the biological pathways influenced by DEPs in both mild and severe psoriatic non-lesional skin versus healthy skin. Cytokines/chemokines, proteases, and protease inhibitors showing at least twofold changes (increase or decrease) in both mild and severe psoriatic non-lesional (PS-NL) vs. healthy (**A**), mild PS-NL-only vs. healthy (**B**), and severe PS-NL-only vs. healthy skin (**C**). For mild psoriasis, the mean Psoriasis Area and Severity Index (PASI) was 9.06, while for severe psoriasis, the mean PASI was 28.93. Each group utilized *n* = 3 pooled protein extracts. Pixel densities of the different proteins were determined by the Image Studio software (LI-COR Biosciences, Lincoln, NE, USA) and presented as relative protein expression. In the visualization, the steel blue color corresponds to the lowest expression, red indicates the highest expression, and white represents medium expression levels of the given protein. Proteins are indicated by gene identifiers and listed in Appendix A Table A1. Green squares label the cytokine/chemokine group, yellow squares indicate proteases, and grey squares mark protease inhibitors. The most significant biological pathways affected by DEPs in both mild and severe PS-NL vs. healthy skin (**D**), in mild PS-NL-only vs. healthy skin (**E**), and in severe PS-NL-only vs. healthy skin (**F**) were identified by the Reactome over-representation test. Results were filtered by statistical significance (*p* ≤ 0.05), and numbering indicates the order of significance. FDR: false discovery rate. Known data for the expression of DEPs in psoriatic lesional and non-lesional skin and their severity-related expression (**G**). The red arrow indicates increased expression, and the blue arrow marks decreased expression. + means known protein expression; – means that no data were found [19,20,21,22,23,24,25,26,27,28,29,30,31,32,33,34,35,36,37,38,39,40]. Comparison of the foldchange direction of DEP array data and microarray data of healthy versus non-lesional psoriatic skin (**H**). Matched foldchange direction is presented. PS-NL: psoriatic non-lesional.

In order to find PS-NL-specific and potential psoriasis-severity-specific changes in non-lesional skin, proteins with at least twofold changes in mild and severe PS-NL versus healthy skin were selected and identified as DEPs. DEPs in both mild and severe PS-NL versus healthy skin showed reduced expression patterns in all cases. No proteases were identified among these proteins, only cytokines, chemokines, and protease inhibitors (Figure 2A). Based on the Reactome over-representation test, platelet degranulation turned out to be the most significant pathway, and DEPs mainly affected hemostasis-related processes (Figure 2B).

The list of DEPs with differential expression in mild PS-NL-only versus healthy skin primarily includes cytokines and chemokines, as well as protease inhibitors and one protease (Figure 2C). All DEPs showed decreased expression. Reactome analysis revealed that these DEPs were mainly related to the TP53-regulated transcription of death receptors and ligands mechanism (Figure 2D).

DEPs with decreased expression in severe PS-NL-only versus healthy skin were also predominantly cytokines and chemokines, along with protease inhibitors (Figure 2E). The most significant pathway affected by these DEPs was the dissolution of fibrin clots. Additionally, immune regulatory pathways were also identified by the Reactome over-representation test (Figure 2F). In our analysis, when comparing severe PS-NL-only to healthy skin, we observed increased expression of DEPs primarily from the cytokine/chemokine and protease groups (Figure 2E). This group of DEPs mostly affected RUNX1-regulated gene expression of megakaryocyte differentiation and platelet function process (Figure 2F).

A manual literature search was performed to explore the already-known information about all identified DEPs. We found data on the expression of DEPs in lesional psoriatic skin for almost half of the DEPs (ANGPT1 [19], ANGPT2 [19], APP [20], BSG [21], CD14 [22], CFD [23], DKK1 [24], DPP4 [25], FGF19 [26], GDF15 [27], IGFBP3 [28], IL13 [29], IL1B [30], MMP2 [31], MPO [32], PI3 [33], SERPINE1 [34], TIMP1 [35], TIMP2 [31], TIMP3 [36], TNFSF5 [36]). However, only a quarter of the DEPs in the literature were identified in non-lesional skin (ANGPT1 [19], ANGPT2 [19], CD14 [22], DKK1 [24], IGFBD3 [28], IL1B [37], MMP2 [31], PI3 [39,40], TIMP1 [35], TIMP2 [31], TIMP3 [36]). Furthermore, no information was found regarding the severity of psoriasis (Figure 2G).

Since there is no available wholescale proteomic data comparing healthy and non-lesional skin proteomes, we collected microarray data for both healthy and psoriatic non-lesional skin across all groups of the identified DEPs. Afterward, the foldchange direction between our protein array and the microarray data was compared. The matched fold change direction of DEPs was 68.75% in the comparison of both mild and severe PS-NL versus healthy skin. It showed a 41.66% agreement with the mild PS-NL-only versus healthy group, and a 73.33% matched foldchange direction was observed in the collation of the severe PS-NL-only versus healthy skin (Figure 2H).

### 2.3. DEPs Between Severe PS-NL Versus Mild PS-NL Skin Mainly Influenced Methyl-CpG-Binding Protein 2 (MECP2)-Regulated Neuronal Ligand Transcription and Extracellular Matrix Degradation

**Figure 3:**
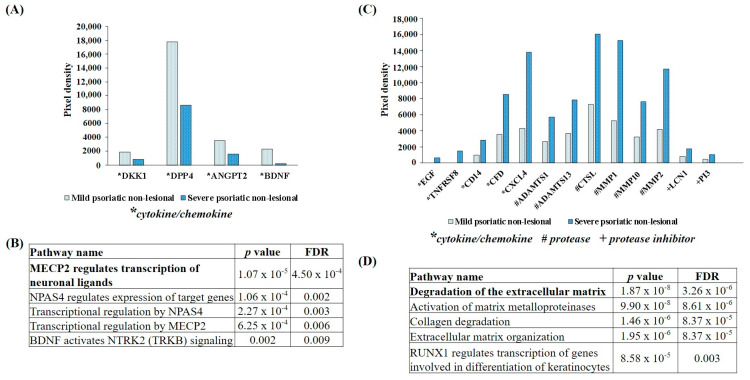
Differentially expressed proteins (DEPs) and the biological pathways affected by DEPs in severe versus mild psoriatic non-lesional skin. DEPs with decreased (**A**) and increased (**B**) expression in the severe psoriatic non-lesional (PS-NL) vs. mild PS-NL skin are presented. Light blue dotted columns indicate mild PS-NL and blue dotted columns mark severe PS-NL pixel densities. For mild psoriasis, the mean Psoriasis Area and Severity Index (PASI) was 9.06, while for severe psoriasis, the mean PASI was 28.93. Each group utilized *n* = 3 pooled protein extracts. Proteins are indicated by gene identifiers and listed in Appendix A Table A1. The most relevant biological pathways affected by decreased (**C**) and increased (**D**) DEPs in the severe vs. mild PS-NL skin were analyzed by the Reactome over-representation test. Results were filtered by statistical significance (*p* ≤ 0.05), and numbering indicates the order of significance. FDR: false discovery rate.

DEPs with a twofold increase or decrease in severe PS-NL versus mild PS-NL skin were also selected and analyzed by the Reactome over-representation test. All DEPs with decreased expression belonged to the cytokine/chemokine group (Figure 3A), and they primarily affected the MECP2-related transcription of neuronal ligands. Furthermore, pathways predominantly affected by the decreased DEPs were primarily related to neuronal gene expression, with all of these DEPs being associated with brain-derived neurotrophic factor (BDNF) (Figure 3B). However, there was a higher proportion of DEPs with increased expression in severe PS-NL versus mild PS-NL skin, which predominantly belonged to protease and cytokine/chemokine groups (Figure 3C). Based on the Reactome test, the degradation of the extracellular matrix was primarily affected (Figure 3D).

### 2.4. Brain-Derived Neurotrophic Factor (BDNF), 5-Methylcytosine (5mC), and 5-Hydroximethylcytosine (5hmC) in the Non-Lesional Epidermis Showed Psoriasis-Severity-Associated Reduced Signal and Nuclear Expression Pattern of MECP2 Was Also Altered in Psoriatic Non-Lesional Versus Healthy Skin

**Figure 4:**
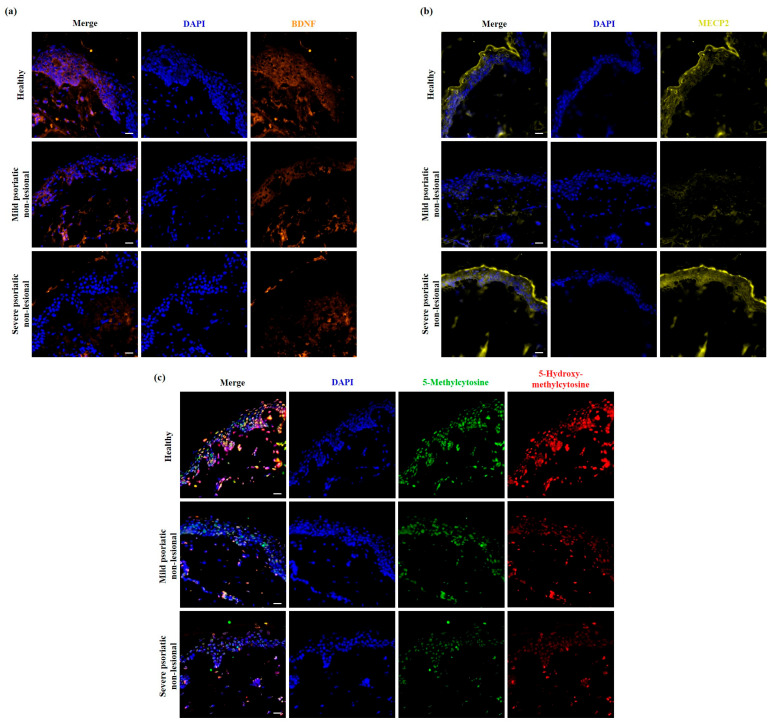
Psoriasis-severity-related reduced expression of brain-derived neurotrophic factor (BDNF), 5-methylcytosine (5mC), and 5-hydroxymethylcytosine (5hmC), and altered expression pattern of methyl-CpG binding protein 2 (MECP2) in psoriatic non-lesional versus healthy skin. Immunofluorescence staining for BDNF (**a**), MECP2 (**b**), as well as 5mC and 5hmC (**c**) in mild and severe psoriatic non-lesional (PS-NL) skin, and healthy skin. Mild psoriatic patients’ Psoriasis Area and Severity Index (PASI) scores were between 5.9 and 11.5; severe psoriatic patients’ PASI scores were between 20.6 and 28.93. Representative images are shown; *n* = 3 donors were used in each group; 40× (oil) original magnification, scale bar: 20 μm; DAPI: 4′,6-diamidino-2-phenylindole; Zeiss AxioImager Z1 microscope (Carl Zeiss AG, Oberkochen, Germany).

BDNF belongs to the neurotrophin family of growth factors and is primarily expressed in the brain but has functions in other organs as well [41]. BDNF, on the one hand, showed decreased expression in both mild and severe PS-NL skin compared to healthy skin, and its level was lower when we compared severe PS-NL with mild PS-NL skin. On the other hand, BDNF-related processes were over-represented based on the analysis. Thus, BDNF was selected for immunofluorescence staining to confirm the obtained results. We detected BDNF-positive cells in both the dermis and epidermis of healthy skin, and BDNF showed disease-severity-related reduced expression in the non-lesional epidermis (Figure 4a), which was in line with the observed differences in BDNF expression based on the array.

BDNF is a known neuronal ligand that is regulated by MECP2 [42]. Among the over-represented BDNF-related pathways, MECP2-associated neuronal ligand transcription was identified by the Reactome test; therefore, we performed MECP2 immunofluorescence staining as well. MECP2 expression was also observed in the cytoplasm and nuclei of dermal and epidermal cells of healthy skin. However, the intensity of the expression was lower in the non-lesional epidermis of patients with mild symptoms, and nuclear MECP2 expression was more characteristic in severe PS-NL epidermis (Figure 4b).

As a methyl-CpG-binding protein, MECP2 can bind to methylated DNA and functions as a DNA methylation reader. The binding of MECP2 to 5mC plays a role in transcription activation, and its binding to 5hmC takes part in the transcription repression of genes, not only neuronal ligand genes [43]. Therefore, we aimed to study whether we could find any psoriasis severity-associated alterations in DNA methylation of the non-lesional skin. A disease severity-related decrease in the staining intensity of 5hmC was observed in the non-lesional versus healthy skin by immunofluorescence staining. Additionally, the expression of 5mC was also clearly reduced when comparing severe PS-NL skin with mild PS-NL and healthy skin (Figure 4c).

### 2.5. Clinically Resolved Psoriatic Skin Has a Distinct Cytokine and Chemokine Expression Profile

**Figure 5:**
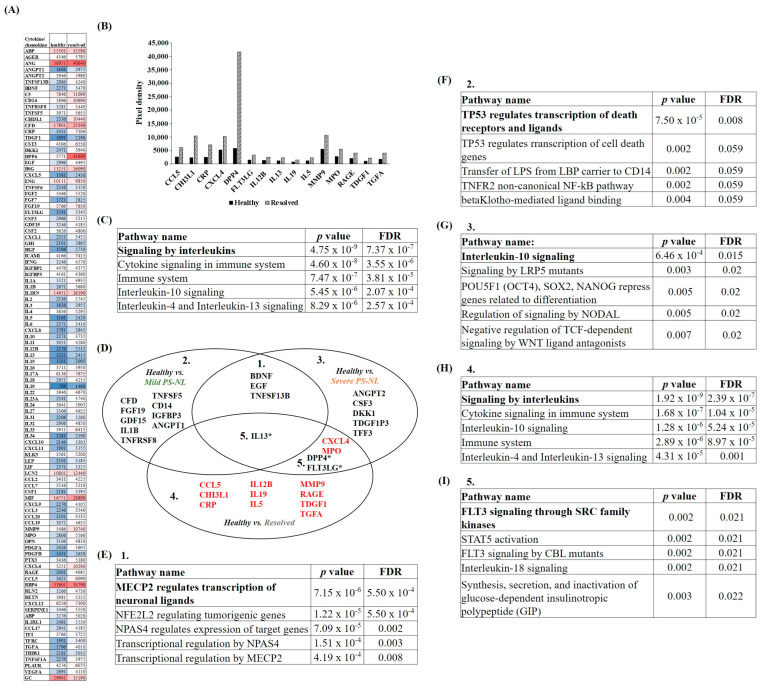
Distinct expression of cytokines and chemokines in clinically resolved psoriatic skin. The levels of cytokines and chemokines were compared in whole skin punch biopsies from healthy donors and resolved psoriatic skin of treated patients (*n* = 3 pooled protein extracts for each group) (**A**). The Image Studio software (LI-COR Biosciences, Lincoln, NE, USA) was used to determine the pixel densities of the different proteins. In the visualization, the blue color corresponds to the lowest expression, red indicates the highest expression, and white represents medium expression levels for the given protein. Differentially expressed cytokines and chemokines in resolved vs. healthy skin are presented by pixel densities (**B**). The black color indicates the healthy group, and the white, dotted pattern marks the resolved group. The most relevant biological pathways affected by these differentially expressed cytokines and chemokines in the resolved vs. healthy skin were analyzed by the Reactome over-representation test (**C**). DEPs of cytokines and chemokines in the mild and severe psoriatic non-lesional (PS-NL) vs. healthy skin and resolved vs. healthy skin were also compared (**D**). Unlabeled proteins showed decreased expression, and red-marked proteins showed increased expression. Protein expression labeled with an asterisk decreased in the mild and severe PS-NL vs. healthy skin and increased in the resolved vs. healthy skin. The Reactome test was performed to reveal the most relevant pathways of differentially expressed cytokines and chemokines 1. in both mild and severe PS-NL vs. healthy skin (**E**); 2. in mild PS-NL-only vs. healthy skin (**F**); 3. in severe PS-NL-only vs. healthy skin (**G**); 4. in resolved-only vs. healthy skin (**H**); and 5. in resolved vs. healthy skin for the *—labeled opposite expression pattern showing differentially expressed cytokines and chemokines (**I**). The Reactome results were filtered by statistical significance (*p* ≤ 0.05), and numbering indicates the order of significance. FDR: false discovery rate. Proteins are indicated by gene identifiers and listed in Appendix A Table A1. PS-NL: psoriatic non-lesional.

Clinically resolved skin looks similar to healthy-looking non-lesional skin of untreated psoriatic patients with either mild or severe symptoms. The expression of cytokines and chemokines were dominant among the DEPs between healthy and psoriatic non-lesional skin of patients with different psoriasis severities. Currently, biological therapies targeting different cytokines offer the most effective treatment options [1]. Since little is known about the cytokine levels in the resolved skin of systemically treated patients, we also compared the expression profiles of cytokines and chemokines of resolved versus healthy skin. In resolved skin, a slightly increased expression pattern of cytokines and chemokines was found compared to healthy skin (Figure 5A), as opposed to mainly decreased expression in mild and severe PS-NL skin.

Differentially expressed cytokines and chemokines (DECs) that showed at least twofold changes in resolved vs. healthy skin were also selected (Figure 5B). These proteins were predominantly associated with the interleukin signaling pathway; additionally, immune regulatory processes were also identified by the Reactome over-representation test (Figure 5C). While DECs in mild and severe PS-NL versus healthy skin showed mostly decreased expression, increased levels of DECs were found in resolved versus healthy skin (Figure 5D).

DECs that showed at least a twofold change were then analyzed separately. MECP2-regulated transcription of neuronal ligands was identified as the most affected pathway by DECs in both mild and severe PS-NL versus healthy skin (Figure 5E). DECs in the mild PS-NL-only versus healthy skin primarily affected the TP53-regulated transcription of cell death receptors and ligands process (Figure 5F). DECs in severe PS-NL-only versus healthy skin predominantly influenced interleukin-10 signaling (Figure 5G). The pathway affected by DECs in resolved skin only compared to healthy skin was the interleukin signaling pathway (Figure 5H). Cytokines and chemokines with twofold higher expression compared to healthy skin were found exclusively in severe PS-NL skin. Notably, these proteins also exhibited a twofold increase in expression in resolved skin. Interestingly, IL-13 decreased twofold in both severe and mild PS-NL versus healthy skin; at the same time, in resolved skin, the expression of IL-13 increased twofold. Similarly, in severe PS-NL skin, the expression of DPP4 and FLT3LG decreased twofold, while in resolved skin, it increased twofold compared to healthy skin. We analyzed these proteins together, and they primarily affected FLT3 signaling through the SCR family kinases pathway (Figure 5I).

### 2.6. A Noticeable Difference in Known Psoriasis-Associated Cytokine and Chemokine Expression Profile Was Observed in Resolved Versus Non-Lesional Skin Compared to Healthy Skin

**Figure 6:**
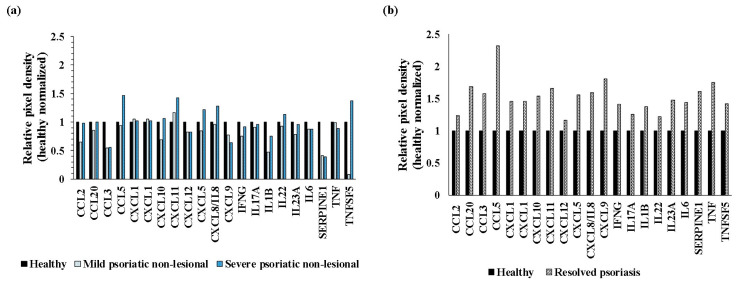
The expression of known psoriasis-related cytokines and chemokines in the mild and severe psoriatic non-lesional versus healthy skin is mainly reduced or non-altered but slightly increased in resolved versus healthy skin. Healthy normalized, relative pixel densities of known psoriasis-related cytokines and chemokines are presented in the mild and severe psoriatic non-lesional (PS-NL) versus healthy skin (**a**) and in resolved psoriatic versus healthy skin (**b**) using our cytokine/chemokine protein array data (*n* = 3 pooled protein extracts for each group). Black color indicates healthy skin, light-blue dotted columns indicate mild PS-NL, blue dotted columns mark severe PS-NL, and white dotted pattern marks the resolved group pixel densities.

As the expression of cytokines and chemokines in psoriatic lesional skin is relatively well-known, and cytokines are the most frequently targeted molecules by biological therapies [1], we selected the known psoriasis-related cytokines and chemokines [1,5,7] and checked their levels in the mild and severe PS-NL and the resolved versus healthy skin. Healthy normalized expression levels of these proteins were mostly detectable at similarly reduced or unchanged levels in mild and severe PS-NL compared to healthy skin (Figure 6a). However, their expression was slightly increased in the resolved versus healthy comparison (Figure 6b).

### 2.7. Nuclear Presence of MECP2 Was Found to Be Increased, While the Staining Patterns of 5mC and 5hmC Were Decreased in Clinically Resolved Psoriatic Skin Compared to Healthy Skin

**Figure 7:**
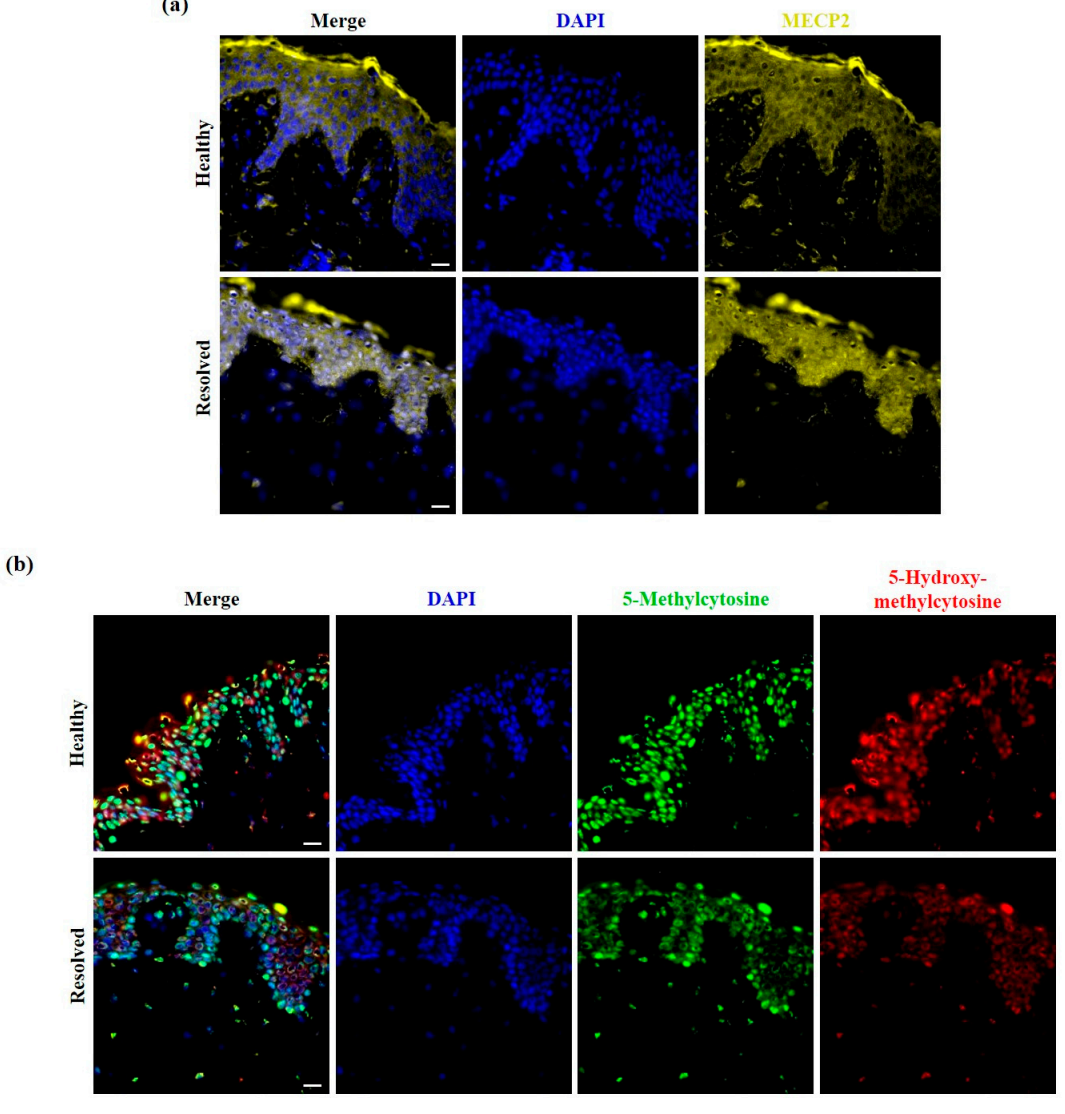
Increased nuclear expression of methyl-CpG binding protein 2 (MECP2) and decreased staining patterns of 5-methylcytosine (5mC) and 5-hydroxymethylcytosine (5hmC) in resolved versus healthy skin. Immunolocalization of MECP2 (**a**) and 5mC and 5hmC (**b**) in psoriatic resolved versus healthy skin. Representative images are shown; *n* = 3 donors were used for each group; 40× (oil) original magnification, scale bar: 20 μm; DAPI: 4′,6-diamidino-2-phenylindole; Zeiss AxioImager Z1 microscope (Carl Zeiss AG, Oberkochen, Germany).

In terms of cytokine and chemokine expression, processes related to MECP2 were identified as key mechanisms that could potentially distinguish psoriatic non-lesional skin from healthy skin and differentiate between non-lesional skin types of varying degrees of severity. We investigated whether the expression patterns of MECP2, 5mC, and 5hmC in resolved skin differed from those in healthy skin. Immunofluorescence staining revealed that MECP2 was more frequently localized in the nucleus of the resolved epidermis (Figure 7a). Furthermore, the staining intensities of 5mC and 5hmC were lower in the resolved epidermis compared to the healthy epidermis (Figure 7b). These findings are consistent with the observations made in severe PS-NL versus healthy skin.

## 3. Discussion

According to the framework hypothesis, the entire psoriatic epidermis has the inherent capacity to manifest clinical lesions [13]. Additionally, the non-lesional skin of patients may exhibit signs of latent psoriasis [4], and protective changes aimed at restraining lesion formation are also observed [14]. Furthermore, stress-related regulation is also altered in the non-lesional skin, and stress proteins, e.g., the cell cycle inhibitor p27, can show severity-related expression in the non-lesional epidermis [17]. There is growing evidence of the role of the tissue environment in the development of psoriatic lesions, which can be regulated by cytokines, chemokines, proteases, and protease inhibitors [1]. In this study, we found that cytokines, chemokines, and proteases showed mainly decreased expression in mild PS-NL skin. However, several proteins in these groups showed increased expression in severe PS-NL skin, indicating severity-specific expression of these proteins. A recent study reported that severe psoriasis can alter the composition of the non-lesional skin [44]. Our study suggests that the increased expression of cytokines, chemokines, and proteases in severe PS-NL skin may be a consequence of disease severity.

The reduced expression levels of protease inhibitors in the psoriatic non-lesional skin suggest changes that are specific to the non-lesional skin, rather than being related to the severity of psoriasis. DEPs in both mild and severe PS-NL skin were mainly protease inhibitors with decreased expression, mostly affecting hemostasis- and platelet-related processes. In addition, pathways affected by DEPs in the severe PS-NL-only group, such as fibrin clot dissolution and RUNX1-regulated platelet functions, were also related to hemostasis. Platelets can contribute to inflammation by producing growth factors, cytokines, and chemokines [45]. They may influence the initiation and progression of immune responses in psoriatic skin and blood vessels, contributing to psoriasis and its associated cardiovascular comorbidities [46]. In psoriasis, inflammatory mediators can cause platelet activation, which may exacerbate the inflammatory response [47]. The mechanisms affected by platelet function highlight the severity-dependent altered inflammatory response of the non-lesional skin and suggest slightly activated inflammation in the non-lesional skin of severe psoriatic patients. In the lesional skin of psoriatic patients, keratinocytes exert resistance to apoptosis, which is dependent on the localization, level, and activation of p53 [48]. However, no significant difference was observed in the number of p53-positive cells in the non-lesional versus healthy epidermis [49]. All of the DEPs in the mild PS-NL-only group showed decreased expression, with a predominance of cytokines/chemokines. These DEPs with decreased expression mainly affected the TP53-related transcription of cell death receptors and ligands, suggesting potential reduced activity for this pathway. Decrease in the transcription of cell death receptors and ligands in mild PS-NL skin can contribute to reduced keratinocyte apoptosis, which is known in lesional keratinocytes and can be observed in mild PS-NL skin.

Based on our literature search, several data are available regarding the lesional skin; however, little is known about data on DEPs in the non-lesional skin, and there is hardly any reported information on their association with psoriasis severity. The matched foldchange direction of DEP array data and microarray data for both mild and severe PS-NL versus healthy skin and severe PS-NL-only versus healthy skin was almost identical and may indicate mainly transcriptional regulation of these proteins. In addition, the lower percentage of matching foldchange direction between DEP array data and microarray data of mild PS-NL-only versus healthy skin (41.66%) suggests a possible severity-specific transcriptional regulation of these proteins. These findings emphasize the significance of considering disease severity when studying the psoriatic non-lesional skin.

According to our array results, the expression of cytokines, chemokines, and proteases can principally distinguish the severe PS-NL skin from the mild PS-NL skin. DEPs with decreased levels in the severe versus mild PS-NL skin mainly influenced the MECP2-related transcription of neuronal ligands. This process and the other most affected mechanisms were also related to the neuronal ligand BDNF among the DEPs. Furthermore, decreased levels of BDNF were detected in both mild and severe PS-NL skin compared to healthy skin. The same process of BDNF-related, MECP2-regulated transcription of neuronal ligands was identified as one of the main pathways affected. However, there is limited data on BDNF and MECP2 in the context of psoriasis. Previous studies have described reduced plasma levels of BDNF in psoriatic patients, with similar levels in both mild and severe cases of psoriasis [50]. Another study reported a negative correlation between BDNF levels and the severity of depression and psoriasis vulgaris [51]. These findings support the brain–skin relationship in psoriasis. However, the expression of BDNF in psoriatic skin has not been previously reported. Immunofluorescence staining revealed decreased BDNF expression in non-lesional skin compared to healthy skin, which was associated with disease severity. This finding supports our array results and suggests that keratinocytes may contribute to the reduced BDNF levels in non-lesional psoriatic skin. Further studies may provide information about the role of BDNF in the non-lesional epidermis. Limited data are available on psoriasis and the DNA methylation reader MECP2. Downregulated expression of MECP2 in peripheral blood mononuclear cells of psoriatic patients has been reported [52], suggesting abnormal DNA methylation in the immune cells of these patients [53]. However, the immunostaining of MECP2 showed reduced expression in the mild PS-NL epidermis and characteristic nuclear presence in the severe PS-NL epidermis. Since MECP2 is involved in epigenetic regulation, facilitating the regulation of gene expression without altering the DNA sequence [54], our findings suggest the possibility of abnormal epigenetic regulation in the non-professional immune cells, the keratinocytes. MECP2 is known to function in the nucleus, but its cytoplasmic localization has also been previously described in endothelial progenitor cells [54] and neuronal cells [55,56]. Similarly, we also identified its nuclear and cytoplasmic expression in the epidermis. Recent studies have shown that epigenetic factors such as DNA methylation, chromatin modifications, and non-coding RNA regulation may significantly impact the development of psoriasis [57]. Several differentially methylated regions [58] and CpG sites were identified in psoriatic skin compared to healthy controls [59,60]. The study found that certain CpGs with differential methylation were significantly enriched in multiple PSORS regions, and they epigenetically regulated the expression of key pathogenic genes [60]. Additionally, the psoriatic epidermis exhibited various types of dysregulated epigenetic changes such as hypermethylation, hypomethylation, and hydroxyl-methylation [61]. A previous study showed substantial differential DNA methylation patterns of non-lesional versus healthy skin, suggesting that DNA methylation contributes to gene expression upon future triggers [62]. In our study, we observed psoriasis-severity-dependent reduced expression of 5mC and 5hmC in the non-lesional epidermis compared to healthy skin. Our research group recently observed that mild and severe inflammation can lead to epigenetic regulation with distinct 5hmC patterns, and decreased 5mC levels in inflamed keratinocytes compared to control keratinocytes [63]. The reduced staining of 5mC and 5hmC in the non-lesional skin compared to healthy epidermis suggests an inflammatory environment in the non-lesional skin that may be dependent on psoriasis severity.

Matrix metalloproteinases play a crucial role in tissue organization in both physiological and pathological conditions. They are involved in tissue remodeling, cell migration, vasodilation, angiogenesis, and regulation of the inflammatory response. These functions are also affected in psoriasis pathogenesis [11]. Our findings suggest that in severe PS-NL, compared to mild PS-NL skin, the DEPs primarily consist of proteases, predominantly associated with ECM degradation-related processes. This indicates an altered tissue organization/homeostasis in the non-lesional skin that is related to the severity of the disease.

In resolved psoriatic skin, not only can tissue-resident memory cells remain in the dermis and epidermis [64], but a disease-residual transcriptomic and epigenetic profile in epidermal keratinocytes has also been described despite the healthy-looking non-lesional state [5]. The cytokine/chemokine expression in resolved skin was predominantly similar or slightly increased compared to healthy skin, which was essentially different from the dominantly reduced levels observed in mild and severe PS-NL versus healthy skin, suggesting disease-residual interleukin signaling in the resolved skin. The most affected pathways based on DECs between mild and severe PS-NL and resolved versus healthy skin revealed newly identified mechanisms; one of them was interleukin-10 signaling in the severe PS-NL-only versus healthy comparison. IL-10 is produced by innate and adaptive immune cells and has anti-inflammatory and immunosuppressive effects [65]. It is expressed at lower levels in psoriatic lesional skin [66]. DECs with decreased expression may indicate reduced IL-10 signaling and decreased anti-inflammatory effects in both lesional and severe PS-NL skin. DECs with opposite expressions in severe PS-NL versus healthy and resolved versus healthy skin primarily affect FLT3 signaling through the SRC family kinases pathway. Mature DCs express high levels of FLT3 (FMS-like tyrosine kinase 3), which is essential for maintaining their functions [67]. FLT3+ DCs are more abundant in psoriatic lesional skin compared to non-lesional skin, and inhibiting FLT3 has been shown to have an anti-psoriatic effect [68]. In severe PS-NL skin, there was a reduction in proteins associated with FLT3 signaling, whereas the resolved skin showed an increase. This suggests opposite regulation for this pathway, potentially resembling patterns observed in both resolved and lesional skin, and may indicate residual immune activation. Moreover, there was a slight elevation in the expression of cytokines and chemokines linked to psoriasis in resolved skin compared to healthy skin. However, this increase was not evident in non-lesional skin sites, suggesting that the expression of psoriasis-characteristic cytokines and chemokines is independent of disease severity in the non-lesional skin. By suspending the therapy, lesional skin-characteristic residual expression levels of cytokines and chemokines may remain.

In addition to the potential severity-dependent epigenetic regulation in the non-lesional skin, the nuclear presence of MECP2 and overall reduced staining patterns for 5mC and 5hmC were observed in the resolved versus healthy skin. Loss of 5hmC in keratinocyte stem cells, and transit-amplifying cells in the psoriatic lesional epidermis contributes to psoriatic lesion formation [69]. The lower levels of DNA methylation in resolved and non-lesional skin could be a sign of inflammation. The severe PS-NL and resolved skin demonstrated similar, predominantly nuclear staining patterns of MECP2, and lower levels of 5mC and 5hmC compared to the healthy epidermis. The resolved skin samples were obtained from psoriatic donors who previously had severe psoriasis and had undergone systemic treatment with biological therapy. The nuclear presence of MECP2 and decreased DNA methylation in severe PS-NL and resolved skin may serve as a compensatory mechanism for the lack of DNA methylation. Future studies may reveal whether the nuclear presence of MECP2 could act as a marker of inflammation or as a prognostic marker of psoriasis severity. Several studies have suggested a role for MECP2 in inflammatory processes. Rett syndrome is associated with mutations in the MECP2 gene, which can lead to dysregulated immunity and chronic subclinical inflammation [70]. Moreover, MECP2 has also been linked to disturbed inflammatory processes in rheumatoid arthritis [71] and neuroinflammation [72]. Para-inflammation, an intermediate state between homeostasis and the classical inflammatory response, can be observed in stressed and dysfunctional tissues and may lead to subsequent chronic inflammation [73]. Our results indicate that epigenetic regulation, which potentially involves MECP2 and DNA methylation, may contribute to the altered inflammatory response of the non-lesional psoriatic skin, which may be a psoriasis-specific, disease-severity-dependent para-inflammatory state.

**Figure 8:**
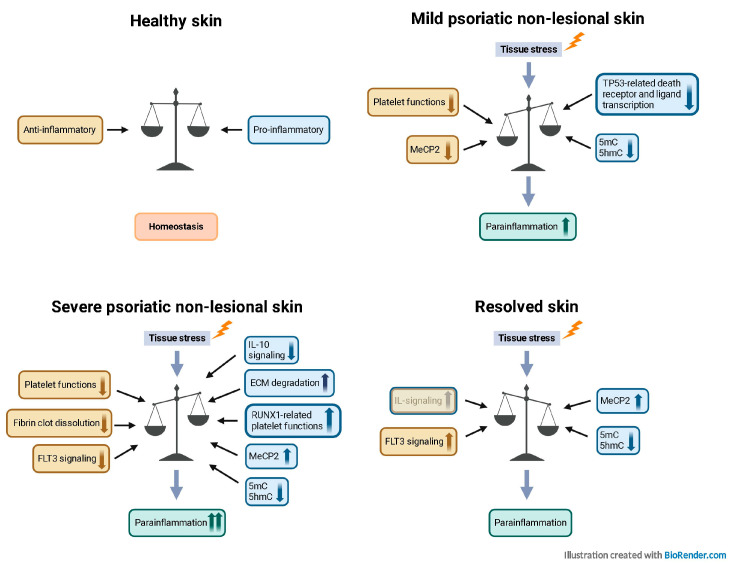
Different stages of inflammation in different healthy-appearing non-lesional skin types. Schematic representation of the identified processes and their potential anti-inflammatory and pro-inflammatory roles. In healthy skin, anti- and pro-inflammatory processes are in a balanced state, providing a homeostatic environment. However, in mild and severe psoriatic non-lesional and resolved psoriatic skin, where tissue stress is present, anti- and pro-inflammatory pathways may alter the balanced homeostatic state, creating a different inflammatory environment with potential distinct, severity-dependent para-inflammatory states. Yellow color indicates potential anti-inflammatory processes, and blue color marks possible pro-inflammatory processes. The illustration was created with BioRender.com.

Our study emphasizes the significance of psoriasis severity when examining the non-lesional skin. The expression of cytokines and chemokines primarily varied according to severity, with noticeable differences observed in clinically resolved skin. The data indicates an altered inflammatory and epigenetic regulation in different non-lesional skin types, with possibly distinct, severity-dependent para-inflammatory states (Figure 8). Despite the apparently healthy phenotype of the different non-lesional skin types, in mild PS-NL skin, the para-inflammation may function with reduced activity in the mild PS-NL skin, and the non-lesional state-maintaining processes may be more active. In severe PS-NL skin, elevated inflammation could be a consequence of the enlarged lesional immune activity on distal non-lesional skin sites. Additionally, disease-residual cytokine signaling is suggested in the resolved skin. Furthermore, differences in epigenetic regulation and tissue organization between the mild and severe non-lesional skin suggest pre-psoriatic mechanisms that are characteristic of patients with severe psoriasis. Additional research may provide a more comprehensive understanding of the epigenetically regulated genes and their potential role in maintaining the distinct uninvolved phenotypes.

## 4. Materials and Methods

### 4.1. Skin Samples and Ethics

Full-thickness punch biopsies (diameter = 6 mm) were obtained from healthy volunteers (*n* = 7, age 20–60 years) and non-lesional skin of untreated patients with mild to severe chronic plaque-type psoriasis (*n* = 13, age 20–60 years, minimum of 6 cm from the lesional region, Psoriasis Area and Severity Index (PASI): 5.4–37.6). Psoriatic patients did not undergo systemic therapy for at least 8 weeks and local therapy for at least 4 weeks. Whole punch biopsies were collected from patients with moderate-to-severe plaque-type psoriasis who received systemic therapy (ustekinumab) for at least 1 year before taking skin samples from the psoriatic previously-lesional, clinically healed, resolved psoriatic skin, where lesions had been resolved for at least 6 months (*n* = 3, age 20–60 years). All skin samples originated from male donors. The study was approved by the Human Investigation Review Board of the University of Szeged (PSO-EDAFN-002, 34/2015, 3517, 23 February 2015, Szeged, Hungary; HCEMM-001, 10/2020, 4702, 20 January 2020, Szeged, Hungary; PSO-CELL-01, 90/2021, 4969, 26 April 2021, Szeged, Hungary). Tissue samples were collected after written informed consent, following the Helsinki Declaration, and used for protein array and immunofluorescence methods.

### 4.2. Protein Extraction from Punch Biopsies

Whole skin punch biopsies of each different donor (healthy volunteers (*n* = 3); non-lesional skin of psoriatic patients with mild disease (mean PASI, 9.06; *n* = 3) and with severe disease (mean PASI, 28.93; *n* = 3)) were cut into small pieces using razor blades and placed in 1% protease inhibitor cocktail and 1% phenylmethylsulfonyl fluoride (Sigma-Aldrich, St. Louis, MO, USA) containing phosphate-buffered saline. The samples were then vortexed with glass beads of different sizes (425–600 µm and 710–1180 µm, Sigma Aldrich, St. Louis, MO, USA) for 3 sets of 5 min each. Triton X-100 (Sigma Aldrich, St. Louis, MO, USA) was added to the samples to a final concentration of 1%. Following this homogenization step, a cycle of freezing (at −80 °C) and thawing (at room temperature) was performed. The samples were subsequently centrifuged to remove tissue debris from the protein extract, and the total protein concentration of the supernatants was measured using the bicinchoninic acid (BCA, Thermo Fisher Scientific, Waltham, MA, USA) assay.

### 4.3. Protein Arrays

Protein extracts from healthy, mild, and severe PS-NL skin were pooled using the same amount of protein from the appropriate protein extracts (*n* = 3 donors from each group). Pooled protein extracts were applied to determine the levels of cytokines/chemokines and proteases/protease inhibitors by Proteome Profiler Human XL Cytokine Array and Proteome Profiler Human Protease/Protease Inhibitor Array kits (R&D Systems, Minneapolis, MN, USA) according to the manufacturer’s instructions. Overall, 99 cytokines/chemokines, 34 proteases, and 32 protease inhibitors were simultaneously detected in duplicate (Appendix A Table A1).

### 4.4. Data Analysis

According to the array’s manufacturer, the pixel densities of the duplicated spots of different proteins on the array membrane were measured using Image Studio software (LI-COR Biosciences, Lincoln, NE, USA). The background signal was subtracted from the mean pixel density values of each protein. The protein expression data were standardized on a per-protein basis using the scale function in base R, and the resulting relative protein expression values were then visualized on a heatmap using the ComplexHeatmap R package (R Studio Software R 4.3.1. R-Studio, Boston, MA, USA). Proteins showing twofold changes (increase or decrease) between the different groups: mild and severe PS-NL versus healthy skin; severe PS-NL versus mild PS-NL; and resolved versus healthy were identified as differentially expressed proteins (DEPs). DEPs were used for the Reactome over-representation test to reveal the pathways most affected by these proteins using the analysis tool in the Reactome database. Gene list analysis was applied and projected to the human organism. Results were filtered by statistical significance (*p* ≤ 0.05) (www.reactome.org accessed on 6 November 2023). Moreover, a manual literature search was performed for DEPs of the mild and severe PS-NL skin to obtain information on known data about the expression of these proteins in psoriatic lesional and non-lesional skin, as well as for potential psoriasis-severity-related alterations. Microarray DEP data were collected from the publicly available database, where data from *n* = 58 psoriatic non-lesional and *n* = 64 healthy whole skin punch biopsies were available independent of gender, age, or disease severity (https://www.ncbi.nlm.nih.gov/geo/query/acc.cgi?acc=GSE13355, accessed on 1 February 2024, ID: GSE13355). The foldchange direction of DEPs was compared between our protein dataset and microarray data.

### 4.5. Immunofluorescence Labeling

Psoriatic and healthy skin-derived biopsies were embedded in a cryogenic solution and then stored at −20 °C until use. Frozen skin sections (6 µm thick) were made and used for immunolabeling. Following incubation with specific labeling reagents, 4′,6-diamidino-2-phenylindole staining (DAPI, Sigma Aldrich, St. Louis, MO, USA) was used for nuclei labeling. The fluorescence signal was observed under a Zeiss Axio Imager Z1 microscope and analyzed with ZEN 2012 Microscope Imaging software (Carl Zeiss AG, Oberkochen, Germany).

#### 4.5.1. BDNF

For BDNF immunostaining, sections were fixed and permeabilized using the Foxp3/Transcription Factor Staining Buffer Set (#50-112-8857, Invitrogen, Thermo Fisher Scientific, Waltham, MA, USA) and blocked with 1% bovine serum albumin (BSA) and 1% normal goat serum (NGS) (both Sigma Aldrich, St. Louis, MO, USA) containing Tris-buffered saline solution (TBS) prepared in the Laboratory from 50 mM Tris-HCl and 150 mM NaCl (both Sigma Aldrich, St. Louis, MO, USA), pH 7.6, at room temperature for 1 h. Mouse anti-human BDNF antibody (1:150, NBP2-37276, Novus Biologicals, Centennial, CO, USA), diluted in 1% NGS containing TBS, was applied on the samples at 4 °C overnight, followed by a 1 h incubation at room temperature with Alexa Fluor 647 conjugated goat anti-mouse IgG (#1856566, 1:500, Life Technologies, Carlsbad, CA, USA) in 1% NGS containing TBS.

#### 4.5.2. MECP2

For MECP2 labeling, sections were fixed with 4% paraformaldehyde and permeabilized with 0.5% TritonX-100 (Sigma Aldrich, St. Louis, MO, USA) containing TBS. This was followed by blocking with 5% NGS and 1% BSA (both Sigma Aldrich, St. Louis, MO, USA) containing TBS at room temperature for 1 h. Rabbit anti-human MECP2 antibody (1:100, NB100-56326, Novus Biologicals, Centennial, CO, USA) diluted in 3% NGS containing TBS was applied on the sections for 16 h at 4 °C. Alexa Fluor 647 conjugated goat anti-rabbit IgG reagent (#1856566, Life Technologies, Carlsbad, CA, USA) was used in 3% NGS containing TBS as the secondary antibody.

##### 5mC and 5hmC

Sections were then preincubated in phosphate-buffered saline (PBS) followed by permeabilization with 0.1% TritonX-100 (Sigma Aldrich, Saint Louis, MI, USA). Then, samples underwent a denaturing procedure that consisted of incubating them with 2 N hydrochloric acid (Sigma Aldrich, Saint Louis, MI, USA) for 1 h. They were then neutralized with 0.1 M Tris-hydrochloric acid (pH 8.3, Sigma Aldrich, Saint Louis, MI, USA). Samples were then blocked with 1% BSA and 1% NGS containing PBS at room temperature for 1 h. Mouse anti-human 5mC (1:500, #A-1014, Epigentek, NY, USA) and rabbit anti-human 5hmC (1:1000, #39769, Active Motif, Carlsbad, CA, USA) antibodies, diluted in 1% NGS containing PBS, were applied on the sections at 4 °C overnight, followed by Alexa Fluor 647 conjugated goat anti-mouse IgG and Alexa Fluor 546 goat anti-rabbit IgG (#1856566 and #1904467, both from Life Technologies, Carlsbad, CA, USA). Mouse IgG1κ (#400102, BioLegend, San Diego, CA, USA) and rabbit IgG (#1793620, Life Technologies, Carlsbad, CA, USA) reagents were used as isotype controls.

## Data Availability

The datasets used and analyzed during the current study are available from the corresponding author on reasonable request.

## Appendix A

**Table A1:** Names of the investigated proteins according to gene identifiers.

Gene ID	Protein Name (Cytokine or Chemokine)
ADIPOQ	Adiponectin/Acrp30
AGER/RAGE	Advanced glycosylation end product-specific receptor/Receptor for advanced glycosylation end products
ANG	Angiogenin
ANGPT1	Angiopoietin-1
ANGPT2	Angiopoietin-2
APOA1	Apolipoprotein A1
BDNF	Brain-derived neurotrophic factor
BSG	Basigin/CD147
C5	Complement Component C5/C5a
CCL17	C-C motif chemokine 17/TARC
CCL19	C-C motif chemokine 19/MIP-3 beta
CCL2	C-C motif chemokine 2/MCP-1
CCL20	C-C motif chemokine 20/MIP-3 alpha
CCL3/CCL4	MIP-1 alpha/beta
CCL5	C-C motif chemokine 5/RANTES
CCL7	C-C motif chemokine 7/MCP-3
CD14	Monocyte differentiation antigen CD14
CD40LG	Tumor necrosis factor receptor superfamily member 5/CD40
CFC1	Cripto-1
CFD	Complement Factor D
CHI3L1	Chitinase 3-like
CRP	C-Reactive Protein
CXCL1	Growth-regulated alpha protein/C-X-C motif chemokine 1
CXCL10	C-X-C motif chemokine 10/IP-10
CXCL11	C-X-C motif chemokine 11
CXCL12	Stromal cell-derived factor 1/SDF-1 alpha/C-X-C motif chemokine 12
CXCL4/PF4	Platelet factor 4/C-X-C motif chemokine 4
CXCL5	C-X-C motif chemokine 5
CXCL9	C-X-C motif chemokine 9/MIG
CSF1	Macrophage colony-stimulating factor 1/M-CSF
CSF2	Granulocyte-macrophage colony-stimulating factor/GM-CSF
CSF3	Granulocyte colony-stimulating factor/G-CSF
CST3	Cystatin C
DKK1	Dickkopf-related protein 1
DPP4	Dipeptidyl peptidase 4/CD26
EGF	Pro-epidermal growth factor
ENG	Endoglin/CD105
FASLG	Fas antigen ligand/Tumor necrosis factor ligand superfamily member 6
FGF19	Fibroblast growth factor 19
FGF2	Fibroblast growth factor 2
FGF7	Fibroblast growth factor 7/KGF
FLT3LG	Fms-related tyrosine kinase 3 ligand
GC	Vitamin D-binding protein
GDF15	Growth/differentiation factor 15
GH1	Somatotropin
ICAM1	Intercellular adhesion molecule 1/CD54
IFNG	Interferon-gamma
IGFBP2	Insulin-like growth factor-binding protein 2
IGFBP3	Insulin-like growth factor-binding protein 3, IBP-3
IL10	Interleukin-10
IL11	Interleukin-11
IL12B	Interleukin-12 subunit beta
IL13	Interleukin-13
IL15	Interleukin-15
IL16	Interleukin-16
IL17A	Interleukin-17A
IL18	Interleukin-18
IL19	Interleukin-19
IL1A	Interleukin-1 alpha
IL1B	Interleukin-1 beta
IL1RL1	Interleukin-1 receptor-like 1/ST2/IL1 R4
IL1RN	Interleukin-1 receptor antagonist protein
IL2	Interleukin-2
IL22	Interleukin-22
IL23A	Interleukin-23 subunit alpha
IL24	Interleukin-24
IL27	Interleukin-27 subunit alpha
IL3	Interleukin-3
IL31	Interleukin-31
IL32	Interleukin-32
IL33	Interleukin-33
IL34	Interleukin-34
IL4	Interleukin-4
IL5	Interleukin-5
IL6	Interleukin-6
IL8	Interleukin-8
KLK3	Prostate-specific antigen/Kallikrein 3/PSA
LCN2	Lipocalin-2/NGAL
LEP	Leptin
LIF	Leukemia inhibitory factor
MET	Hepatocyte growth factor receptor/HGF receptor
MIF	Macrophage migration inhibitory factor
MMP9	Matrix metalloproteinase-9
MPO	Myeloperoxidase
OPN	Osteopontin
PDGFA	Platelet-derived growth factor subunit A/PDGF-AA
PDGFA/PDGFB	Platelet-derived growth factor subunit A/Platelet-derived growth factor subunit B
PECAM1	Platelet endothelial cell adhesion molecule/CD31
PLAUR	Urokinase plasminogen activator surface receptor/uPAR
PTX3	Pentraxin 3/TSF-14
RBP4	Retinol-binding protein 4
RETN	Resistin
RLN2	Relaxin-2
SERPINE1	Plasminogen activator inhibitor 1/Serpin E1/PAI-1
SHBG	Sex hormone-binding globulin
TFF3	Trefoil factor 3
TFRC	Transferrin receptor protein 1
TGFA	Protransforming growth factor alpha
THBS1	Thrombospondin-1
TIMELESS	Protein timeless homolog/TIM-1
TNF	Tumor necrosis factor/TNF-alpha
TNFRSF9	Tumor necrosis factor receptor superfamily member 8/Lymphocyte activation antigen CD30
TNFSF13B	Tumor necrosis factor ligand superfamily member 13B
VCAM1	Vascular cell adhesion protein 1
VEGFA	Vascular endothelial growth factor A, long form
gene ID	protein name (protease)
ADAM8	Disintegrin and metalloproteinase domain-containing protein 8
ADAM9	Disintegrin and metalloproteinase domain-containing protein 9
ADAMTS1	A disintegrin and metalloproteinase with thrombospondin motifs 1
ADAMTS13	A disintegrin and metalloproteinase with thrombospondin motifs 13
CTSA	Lysosomal protective protein/Cathepsin A
CTSB	Cathepsin B
CTSC	Dipeptidyl peptidase 1/Cathepsin C
CTSD	Cathepsin D
CTSE	Cathepsin E
CTSL1	Procathepsin L/Cathepsin L
CTSS	Cathepsin S
CTSV	Cathepsin L2/Cathepsin V
CTSZ	Cathepsin X/Z/P
DPP4	Dipeptidyl peptidase 4/CD26
KLK10	Kallikrein 10
KLK11	Kallikrein 11
KLK13	Kallikrein 13
KLK3	Prostate-specific antigen/Kallikrein 3/PSA
KLK5	Kallikrein 5
KLK6	Kallikrein 6
KLK7	Kallikrein 7
MME	Neprilysin/CD10
MMP1	Interstitial collagenase/Matrix metalloproteinase-1
MMP10	Stromelysin-2/Matrix metalloproteinase-10
MMP12	Macrophage metalloelastase/Matrix metalloproteinase-12
MMP13	Collagenase 3/Matrix metalloproteinase-13
MMP2	72 kDa type IV collagenase/Matrix metalloproteinase-2
MMP3	Stromelysin-1/Matrix metalloproteinase-3
MMP7	Matrilysin/Matrix metalloproteinase-7
MMP8	Neutrophil collagenase/Matrix metalloproteinase-8
MMP9	Matrix metalloproteinase-9
PCSK9	Proprotein convertase subtilisin/kexin type 9
PI3	Trappin-2/Elafin
PLAU	Urokinase-type plasminogen activator/U-plasminogen activator
PRTN3	Myeloblastin/Proteinase 3
PSEN1	Presenilin-1
gene ID	protein name (protease inhibitor)
AGT	Angiotensinogen/Serpin A8
APP	Amyloid-beta precursor protein
BSG	Basigin/CD147
CST3	Cystatin C
CST6	Cystatin E/M
CSTA	Cystatin A
CSTB	Cystatin B
FETUB	Fetuin B
LCN1	Lipocalin-1
LCN2	Lipocalin-2/NGAL
LXN	Latexin
RECK	Reversion-inducing cysteine-rich protein with Kazal motifs/RECK protein
SERPINA12	Serpin A12
SERPINA5	Serpin A5
SERPINA9	Serpin A9/Centerin
SERPINB5	Serpin B5/Maspin
SERPINB6	Serpin B6
SERPINB8	Serpin B8
SERPINE1	Serpin E1/Plasminogen activator inhibitor 1
SERPINF1	Serpin F1/Pigment epithelium-derived factor
SPINT1	Kunitz-type protease inhibitor 1/HAI-1
SPINT1	Kunitz-type protease inhibitor 1/HAI-1
SPINT2	Kunitz-type protease inhibitor 2/HAI-2
SPINT2	Kunitz-type protease inhibitor 2/HAI-2
SPOCK1	Testican 1/SPOCK1
SPOCK2	Testican 2/SPOCK2
TFPI	Tissue factor pathway inhibitor
TFPI2	Tissue factor pathway inhibitor 2
TIMP1	Metalloproteinase inhibitor 1/TIMP-1
TIMP2	Metalloproteinase inhibitor 2/TIMP-2
TIMP3	Metalloproteinase inhibitor 3/TIMP-3
TIMP4	Metalloproteinase inhibitor 4/TIMP-4
WFDC2	WAP four-disulfide core domain protein 2/HE4
